# P-1193. Activity of isavuconazole against contemporary Mucorales from a worldwide surveillance program

**DOI:** 10.1093/ofid/ofaf695.1386

**Published:** 2026-01-11

**Authors:** Marisa Winkler, Samuel Edeker, Abby Klauer, Paul Rhomberg, Mariana Castanheira

**Affiliations:** Element Materials Technology/Jones Microbiology Institute, North Liberty, Iowa; Element Materials Technology/Jones Microbiology Institute, North Liberty, Iowa; JMI Laboratories, North Liberty, Iowa; Element Materials Technology/Jones Microbiology Institute, North Liberty, Iowa; Element, North Liberty, IA

## Abstract

**Background:**

Isavuconazole (ISA) is the only US FDA-approved antifungal agent with labeling for the treatment of invasive mucormycosis (IM). The prevalence of IM has risen due to the increased use of immunosuppressive agents and conditions which increase the risk of IM. Liposomal amphotericin B (AMB) is typically first-line empirical treatment for IM but has increased toxicity compared to ISA and is only available in an intravenous (IV) formulation while ISA is available in both oral and IV delivery.

**Figure:**
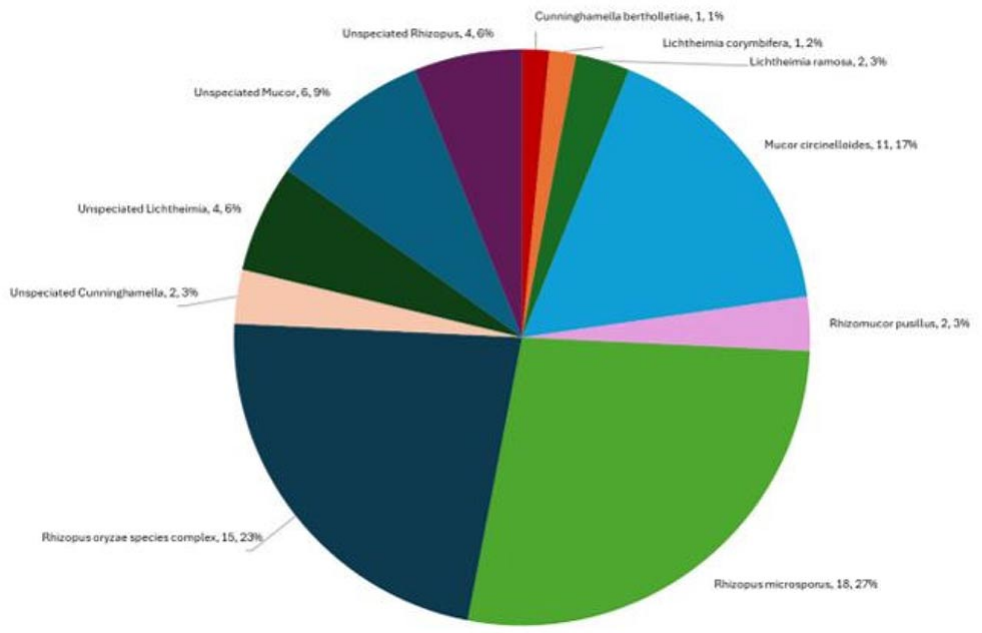
Mucorales species represented in surveillance testing

**Figure:**
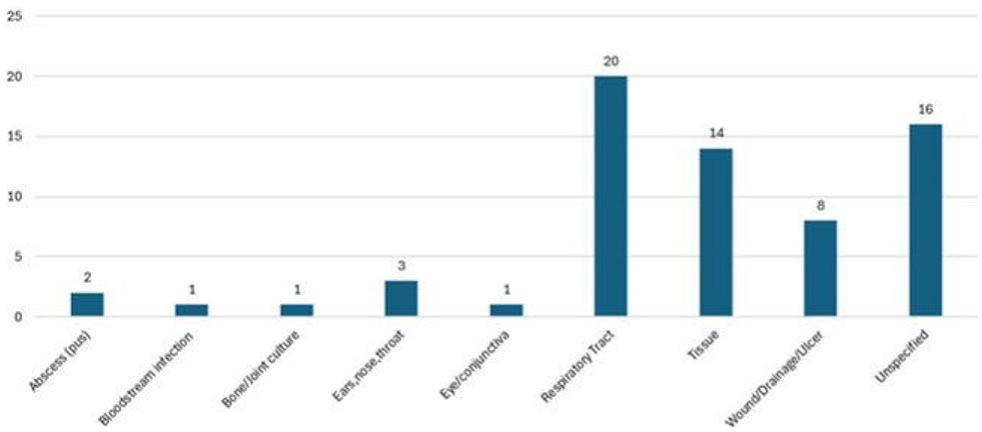
Infection source for collected Mucorales isolates

**Methods:**

A total of 66 Mucorales isolates from invasive fungal infections collected at 27 hospitals in 13 countries between 2021 - 2024 were tested. Confirmatory identification was performed by MALDI or 28S and ITS sequencing. Isolates were tested by reference broth microdilution method according to CLSI guidelines against voriconazole (VRC), posaconazole (POS), ISA, and AMB. No CLSI breakpoints or epidemiological cutoff values are available for interpretation of MICs.

**Figure:**
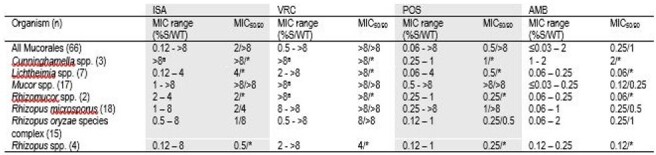
MIC range (mg/L), MIC50/90 (mg/L), for isavuconazole and comparator agents for all tested Mucorales ISA, isavuconazole; VRC, voriconazole; POS, posaconazole; AMB, amphotericin B *MIC50 only as < 10 organisms represented ^a^No range as all organisms at same MIC

**Results:**

11 different organism groups were represented (Figure 1). Infection source was varied (Figure 2). Across all Mucorales, ISA MICs ranged from 0.12 - > 8 mg/L with MIC_50_ 2 mg/L, which was lower than that of VRC and comparable to POS (within 2 doubling dilutions, Table 1). When analyzing individual genera, lower MICs were observed for ISA and POS against *Lichtheimia* (100% ≤ 4mg/L)*, Rhizomucor* (100% ≤ 4mg/L), and *Rhizopus* spp. (89.2% and 91.9% ≤ 4mg/L, respectively). However, higher MICs were observed for ISA and POS against *Mucor* spp. (23.5% ≤ 4mg/L). AMB MICs were ≤ 2 mg/L across all genera.

**Conclusion:**

ISA and POS have low MICs against *Lichtheimia*, *Rhizomucor*, and *Rhizopus* spp. isolates collected from IM infections worldwide between 2021 and 2024. Higher MICs are observed for *Mucor* spp. Given its FDA indication, delivery mechanisms, side-effect profile and drug-drug interactions, ISA is often preferred for long-term treatment of IM compared to AMB or POS. These data support the use of ISA for treatment of IM if *Lichtheimia*, *Rhizomucor*, or *Rhizopus* spp. are identified as the causative organism but waiting for MIC results if *Mucor* spp. is identified.

**Disclosures:**

Marisa Winkler, MD, PhD, Basilea: Advisor/Consultant|Basilea: Grant/Research Support|GSK: Advisor/Consultant|GSK: Grant/Research Support|Melinta Therapeutics: Advisor/Consultant|Melinta Therapeutics: Grant/Research Support|Mundipharma: Advisor/Consultant|Mundipharma: Grant/Research Support|Pfizer: Advisor/Consultant|Pfizer: Grant/Research Support|Pulmocide: Advisor/Consultant|Pulmocide: Grant/Research Support Mariana Castanheira, PhD, Melinta Therapeutics: Advisor/Consultant|Melinta Therapeutics: Grant/Research Support

